# Synthesis, structure, and antioxidant activity of methoxy- and hydroxyl-substituted 2'-aminochalcones

**DOI:** 10.1007/s00706-016-1812-9

**Published:** 2016-08-26

**Authors:** Chiara Sulpizio, Alexander Roller, Gerald Giester, Annette Rompel

**Affiliations:** 1 Fakultät für Chemie, Institut für Biophysikalische Chemie, Universität Wien, Althanstraße 14, 1090 Vienna, Austria; 2 Fakultät für Chemie, Institut für Anorganische Chemie, Universität Wien, Währinger Straße 42, 1090 Vienna, Austria; 3 Fakultät für Geowissenschaften, Geographie und Astronomie, Institut für Mineralogie und Kristallographie, Universität Wien, Althanstraße 14, 1090 Vienna, Austria

**Keywords:** Antioxidant activity, Claisen condensation, Structure activity relationship, X-ray structure determination

## Abstract

**Abstract:**

Three 2'-aminochalcone derivatives (*E*)-1-(2-aminophenyl)-3-(4-hydroxyphenyl)prop-2-en-1-one, (*E*)-1-(2-aminophenyl)-3-(3,4-dihydroxyphenyl)prop-2-en-1-one, and (*E*)-1-(2-amino-4,5-dimethoxyphenyl)-3-(4-methoxyphenyl)prop-2-en-1-one, have been synthesized, characterized, and tested in vitro in order to assess their antioxidant activity. All compounds were characterized on the basis of ^1^H NMR, ^13^C NMR, ESI-mass spectrometry, FT-IR, UV/Vis, and elemental analysis. The X-ray crystal structures of (*E*)-1-(2-aminophenyl)-3-(4-hydroxyphenyl)prop-2-en-1-one and (*E*)-1-(2-amino-4,5-dimethoxyphenyl)-3-(4-methoxyphenyl)prop-2-en-1-one were successfully determined showing a planar molecule geometry. Studies on the biological properties including test of free radical scavenging ability (DPPH test) and superoxide dismutase mimetic activity were performed. The results indicate that the aminochalcone carrying two hydroxyl functionalities in adjacent *meta* and *para* position exhibits a stronger antioxidant activity than the other derivatives.

**Graphical abstract:**

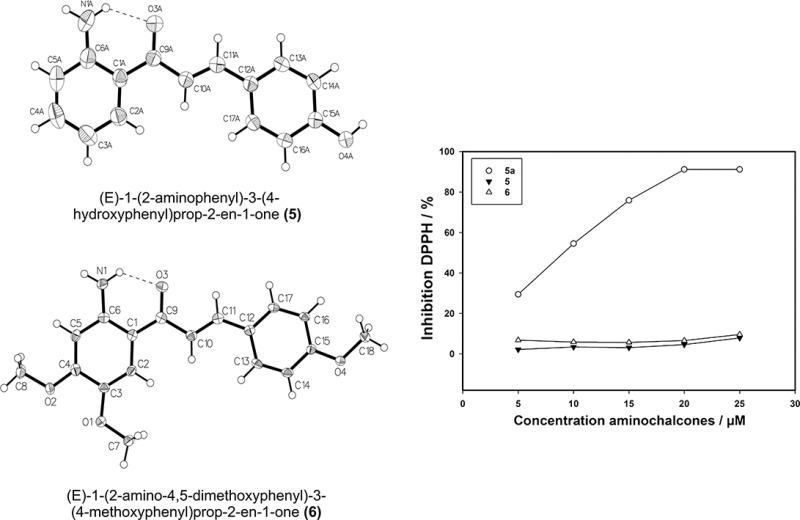

**Electronic supplementary material:**

The online version of this article (doi:10.1007/s00706-016-1812-9) contains supplementary material, which is available to authorized users.

## Introduction

Medicinal plants have always represented one of the most excellent and ancient sources of pharmaceutical agents in the history of medicinal chemistry. Among them chalcones are natural colorful pigments present in several medicinal and edible plants [1]. They act as intermediates in the flavonoid biosynthesis serving different physiological functions such as UV photoprotectors, insect repellents, and attractants of pollinators [2, 3]. Besides these physiological aspects chalcones provide an attractive scaffold for medicinal chemistry, as they possess a wide range of biological activities and are compounds of high therapeutical interest [4].

In the past decades a large number of reports have been published on the pharmacological effects of chalcones, and it has repeatedly been claimed that this natural product is efficient and safe for the prevention and treatment of several diseases such as atherosclerosis and neurodegenerative disorders like Alzheimer disease, preventing the low density lipoprotein (LDL) oxidation [5, 6]. Chalcones have been investigated for their anti-tumor [7], anti-inflammatory [8], antifungal and antibacterial properties [9] as well as for their radical scavenging potential [10]. Structurally they possess a 1,3-diaryl-2-propen-1-one skeleton (see Scheme 1). The presence of the α,β-unsaturated keto functionality (2-propen-1-one chain) seems to be responsible for the medicinal properties of the chalcones [11, 12].

**Figure:**
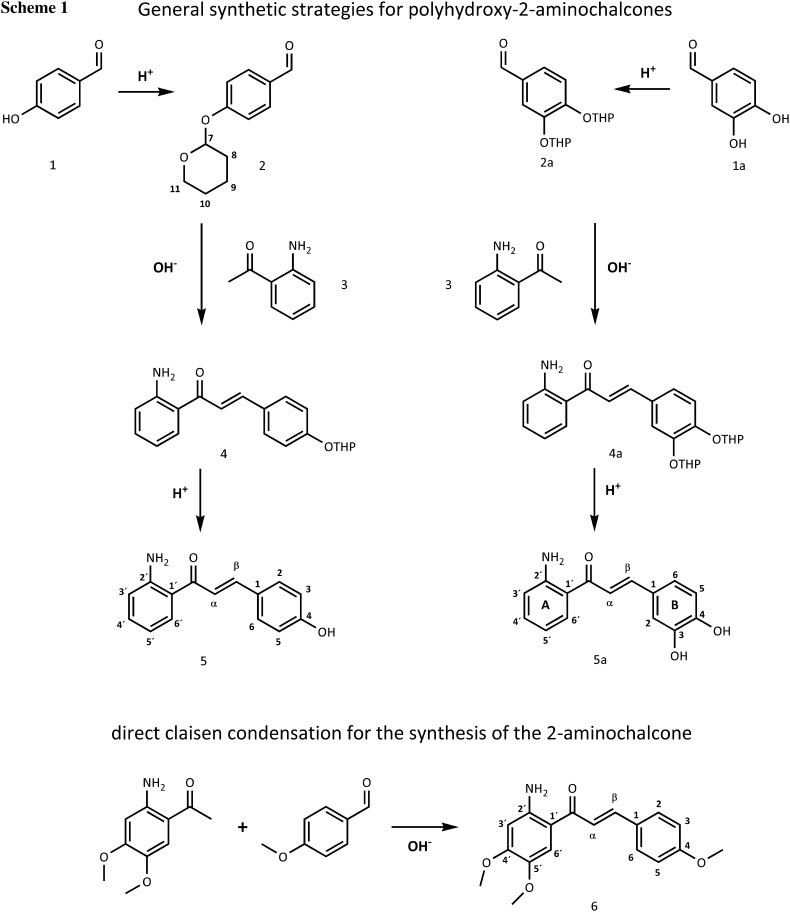

In fact it has been proven in previous studies that the electrophilicity of this α,β-unsaturated carbonyl moiety is involved in the antioxidant [13, 14] activities of chalcones. Additionally, although numerous antioxidant substances belong to different chemical classes, they have a certain electronic and steric characteristic in common: in particular an important requisite would be a planar structure with two hydrophobic moieties (aromatic groups) and an acidic proton (the phenolic proton) [15]. These characteristics, which are all present in chalcones, make them suitable candidates for the design of new drugs; moreover, depending on the substitution pattern of the two rings they can display different spectra of activity. In this work we focused our attention on hydroxyl, methoxyl, and amino substitution in order to observe how they impact the biological activities of chalcones.

Methoxylated and hydroxylated chalcones are also well known for their powerful antioxidant activities. In fact it has been reported in literature that natural chalcones carrying methoxyl and hydroxyl substitutions such as butein, isoliquiritigenin, cardamonin, flavokawain A and B, are able to scavenge reactive oxygen species (ROS) [10]. Normally living organisms are protected against highly reactive oxygen species by an endogenous system of enzymes like superoxide dismutase or other naturally occurring antioxidants widely distributed in the biological system such as ascorbic acid or vitamin E for instance [16]. In fact a high level of ROS can attack essential biological molecules like lipids, proteins, and DNA, and it has been already demonstrated that in case of disease the production of ROS is increased [17]. As a consequence, natural and synthetic molecules possessing multifunctional antioxidant activities such as flavonoids are of great interest and important in disease prevention and in therapy [18]. Within this polyhedral class of compounds, 2'-aminochalcone derivatives still represent a new domain of investigation. Currently they have been investigated for their antitumor activity [19, 20], and additionally they have shown to be useful as photoprotectors in sunscreen formulations as well as in textile polymers or fibers because of their high UV–visible extinction coefficient [21, 22]. Apart from these promising aspects, little is known about the reactivity/response of aminochalcones towards reactive oxygen species. Thus, an evaluation of the potential biological applications of the novel amino chalcone-based compounds will give more insights into their mode-of-action. Therefore, they could represent in the future new templates for novel antioxidants.

In this work three new chalcones with methoxyl, hydroxyl, and amino substitutions on ring A as well as on ring B were synthesized and characterized with the aim to investigate their antioxidant properties and clarify their molecular structure. The compounds are therefore characterized via ^1^H NMR, ^13^C NMR, FT-IR, and UV–Vis and elemental analysis. The crystal structures of the newly synthesized molecules 1-(2-aminophenyl)-3-(4-hydroxyphenyl)prop-2-en-1-one (**5**) and 1-(2-amino-4,5-dimethoxyphenyl)-3-(4-methoxyphenyl)prop-2-en-1-one (**6**) (see Scheme 1) were solved by single-crystal X-ray diffraction in order to study the conformation of the typical chalcones.

## Results and discussion

### Synthesis of 2'-aminochalcone derivatives

All three final compounds (*E*)-1-(2-aminophenyl)-3-(4-hydroxyphenyl)prop-2-en-1-one (**5**), (*E*)-1-(2-aminophenyl)-3-(3,4-dihydroxyphenyl)prop-2-en-1-one (**5a**), and (*E*)-1-(2-amino-4-methoxyphenyl)-3-(3,4-dimethoxyphenyl)prop-2-en-1-one (**6**) were obtained with moderate-to-good yields and purely in the *E* form confirmed by the means of ^1^H NMR and X-ray crystallography. The amino chalcones **5** and **5a** were synthesized according to the most common base-catalyzed Claisen Schmidt condensation using barium hydroxide octahydrate [23]. **5** and **5a** were obtained via a three-step procedure (see Scheme 1). First the appropriate benzaldehydes **1** and **1a** were protected with tetrahydropyranyl (THP) to obtain **2** and **2a**. THP has been chosen as a protecting group because it is easy to insert and remove and it is also stable under normal alkaline coupling conditions [24]. Protection proceeded in both cases in good yield (89 % for **2** and 69 % for **2a**). Subsequently a base-catalyzed aldol condensation reaction between the aminoacetophenone with protected benzaldehydes **2** and **2a** was performed to furnish the protected aminochalcones **4** and **4a** species with a yield of 39 % and 40 %, respectively. Compound **4a** was immediately used without any further purification and its identity checked by ESI-mass spectrometry (see “Experimental” section compound **4a**). Compound **4** instead was purified by flash column chromatography. After purification **4** decomposes very fast turning from yellow to dark red, therefore ^13^C NMR measurements have not been recorded and **4** was immediately used for the condensation step. Finally deprotection was performed to give the hydroxy-2'-aminochalcones **5** and **5a** with a yield of 25 % for **5** and 20 % for **5a**. All the condensation reactions worked only moderately, and purification with liquid column chromatography is challenging after condensation as well as after the hydrolysis reaction. This may be due to the formation of the cyclization side product between the amino group on the A ring and the aromatic B ring, which is very difficult to separate from the target compound by flash column chromatography as the two molecules have the same retention time. The cyclization of 2'-aminochalcones has been reported before [25]. Moreover, the different attempts pointed out that both time and temperature influence the hydrolysis reaction.

Synthesis of the 2'-aminochalcone **6** was carried out between the 2-aminoacetophenone and the 4-methoxybenzaldehyde in one direct reaction leading to a yield of 40 % of the pure product.

### Spectroscopic and mass characterization

Characterization of the synthesized compounds was performed by ESI-mass spectrometry, FT-IR spectroscopy, UV–Vis spectroscopy, ^1^H NMR, and ^13^C NMR. All techniques confirm unambiguously the structure of the final products **5**, **5a**, and **6**. ESI-MS analysis shows the molecular ion peak always with good intensity and in accordance with the calculated empirical formula. The same is valid for elemental analysis results. For compounds **5** and **6** the typical strong NH_2_ asymmetric and symmetric vibrations were visible at 3340/3042 cm^−1^ (**5**) and 3386/3267 cm^−1^ (**6**), respectively. For compound **5a** the NH_2_ stretching overlapped with the strong broad band of the two OH groups [26]. All the absorptions arising from the aromatic rings appear between 1400 and 1600 cm^−1^. The UV–Vis absorption occurs in the range from 480 to 260 nm, showing maxima of absorption at 240, 342, and 399 nm for **5**, 364 and 487 nm for **5a**, and 244, 323, and 412 nm for **6** [27]. These values are attributed to *π*–π* and *n*–π* transitions due to the excitation in the aromatic ring and the C=O group [28].

Finally, the characteristic high value of the coupling constant of the double bond of the enone moiety (see also ^1^H NMR of and Hα and Hβ, “Experimental” section) indicates that the chalcones **5**, **5a**, and **6** were obtained in the *E* form [29]. The Hβ of the olefinic system is more deshielded then Hα because of the π-bond delocalization, and therefore they both appear in the aromatic region. This result is in good agreement with the X-ray structure analyses data obtained for **5** and **6**.

### X-ray crystallography

**Fig. 1:**
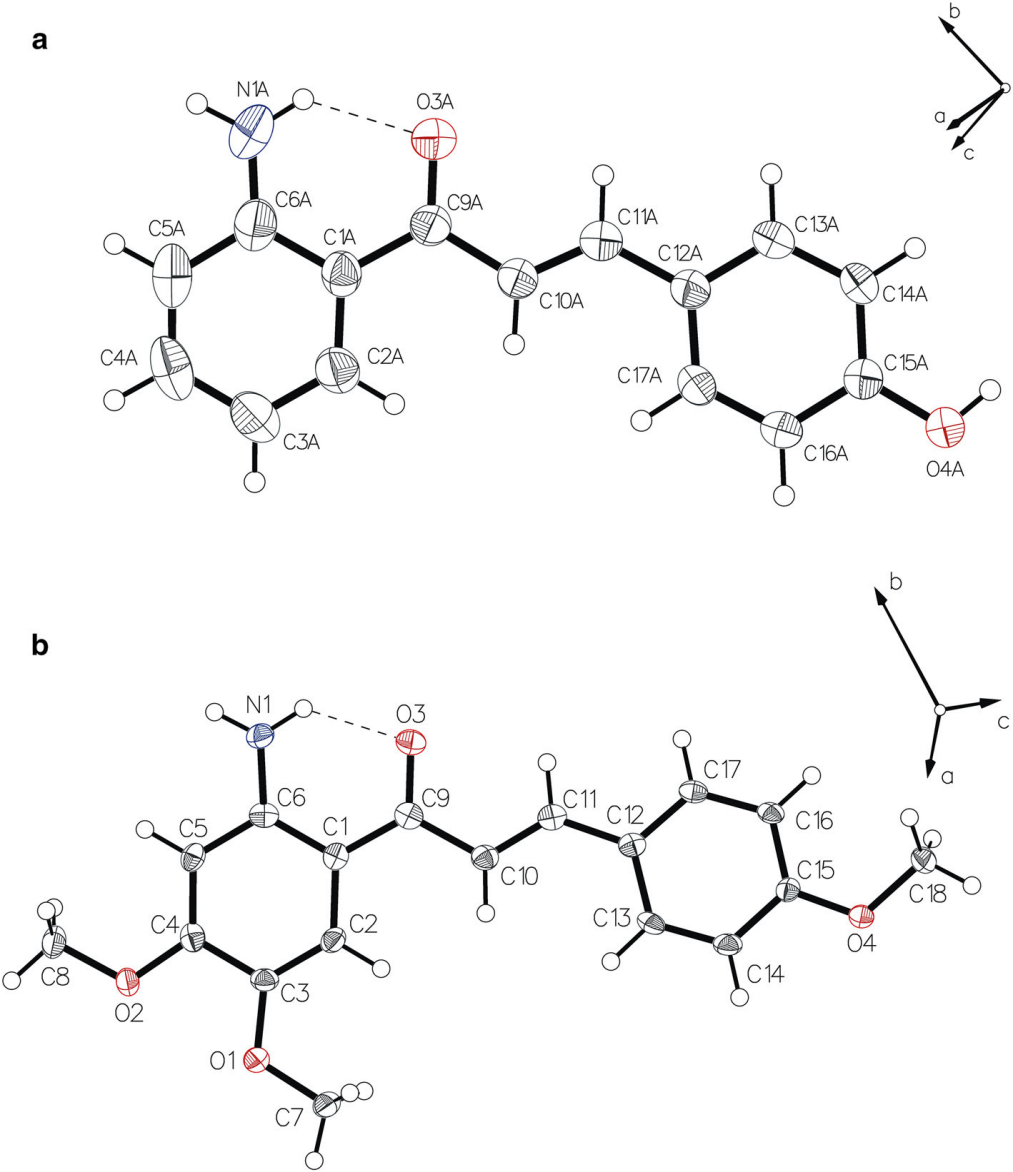
ORTEP style drawings based on X-ray structure analysis of **5** (**a**) and **6** (**b**) with atom numbering scheme. Thermal parameters enclose 50 % probability

**5** and **6** were crystallized at room temperature from a mixture of dichloromethane/methanol 9:5 for **5** and hexane/ethyl acetate 9:2 for **6**. A summary of crystal parameters and refinement details of **5** and **6** are given in Table 1. Compounds **5** and **6** crystallize in the space groups *P2*_*1*_*/c* and *Iba2*, respectively. The asymmetric unit of compound **5** contains five independent molecules, whereas the asymmetric unit of **6** contains one molecule. All bond distances and angles are comparable to previous reports [30] and similar experimental value were previously found for the 2'-aminochalcone (*E*)-1-(2-aminophenyl)-3-(pyridine-4-yl)prop-2-en-1-one [31] and (*E*)-1-(4-aminophenyl)-3-(2,4,5-trimethoxyphenyl)prop-2-en-1-one [32]. The ORTEP diagrams of **5** and **6** prepared with OLEX2 are shown in Fig. 1 along with the atom numbering scheme. The results confirm that the olefinic double bond in both compounds is in the *E* configuration and that the OH group in **5** and the OCH_3_ group in **6** are located in *para* position on ring B. The most interesting structural feature is a plane molecular conformation: in fact the hydrogen bonding interaction between the amino function NH_2_ and the carbonylic O atom seems to play an important role in defining the planar conformation of the molecule. The oxygen atoms O(3A) in **5** and O(3) in **6** act as an acceptor in a moderate intramolecular hydrogen bond involved with the adjacent NA(1)–H_2_ and N(1)–H_2_ functionalities, respectively (see Fig. S1 and S2). The same O(3A) atom in **5** acts again as an acceptor in a likewise moderate intermolecular hydrogen bond interaction with the O(4)–H group. The N(1A)–O(3) and O(3)–O(4) distances are 2.621 and 2.640 Å for compound **5** (see Table 2). Similar distances are observed for compound **6** (Table 3). The intramolecular hydrogen bond seems to be the most significant coadjuvant to the molecular packing forces that support the planarity of the entire molecule, although further contribution could originate also from weak aromatic ring-stacking interactions between the phenyl rings and the keto-enolic moiety.

**Table 1:** Crystal data and structure refinement for compounds** 5** and** 6**

Identification code	**5**	**6**
Empirical formula	C_15_H_13_NO_2_	C_18_H_19_NO_4_
*M*_r_/g cm^−3^	1.305	1.314
Space group	*P2*_*1*_ */c*	*Iba2*
Crystal system	Monoclinic	Orthorhombic
*a*/Å	18.877(4)	13.1460(13)
*b*/Å	16.644(3)	29.598(3)
*c*/Å	19.387(4)	8.1392(6)
*α*/°	90	90
*β*/°	91.781(10)	90
*γ*/°	90	90
*V*/Å^3^	6088(2)	3166.9(5)
*Z*	20	8
*μ*/mm^−1^	0.087	0.093
Reflns collected	126774	17995
Independent reflns	15117	2924
*R*(int)	0.0638	0.0776
GooF on *F* ^2^	1.025	1.033
*R*_1_ [*I* > 2*σ*(*I*)]	0.0579	0.0403
w*R*_2_ (all data)	0.1596	0.0943

**Table 2:** Selected hydrogen bond distances/Å and angles/° for **5**

D	H	A	d(D-H)/Å	d(H-A)/Å	d(D-A)/Å	D-H-A/°
O(4A)	H(4A)	O(3B)^a^	0.84	1.80	2.640(2)	174.0
N(1A)	H(1AB)	O(3A)	0.88	1.96	2.621(3)	130.3

^a^+*X*, 1/2 − *Y*, −1/2 + *Z*

**Table 3:** Selected hydrogen bond distances/Å and angles/° for **6**

D	H	A	d(D-H)/Å	d(H-A)/Å	d(D-A)/Å	D-H-A/°
N(1)	H(1A)	O(3)	0.88	1.93	2.584(4)	130.4
N(1)	H(1B)	O(3)^a^	0.88	2.03	2.890(4)	166.8

^a^+X,1-Y,-1/2+Z

### Antioxidant activity

The antioxidant activity under in vitro conditions has been explored through two different methods, namely the superoxide dismutase (SOD) assay and the DPPH radical scavenging method. The DPPH test relies on the antioxidant ability to quench the radical by hydrogen donation capacity, whereas the SOD-like assay is based on the fact that antioxidants can suppress the formation of reactive oxygen species by inhibiting the enzymes involved in its generation and have superoxide scavenging activities themselves [33, 34]. Both methods have been chosen because they have already been widely used to determine the antioxidant activity of isoflavonoids [35].

### Free radical scavenging ability

**Fig. 2:**
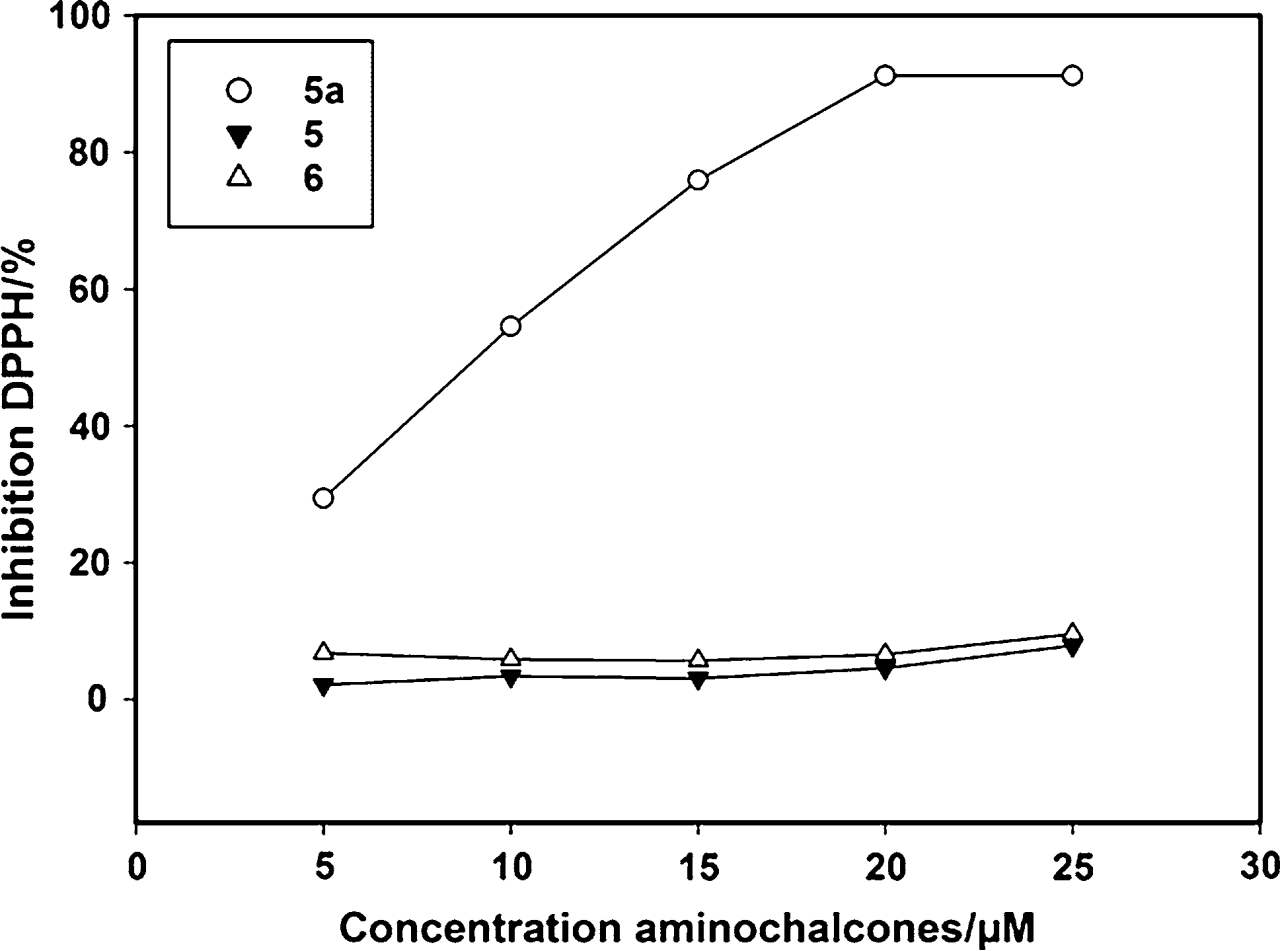
The percentage of inhibition of the free DPPH radical in the presence of **5**, **5a**, and **6**. The concentration of the different compounds is expressed as µM

The free radical scavenging ability of the synthesized 2'-aminochalcones was evaluated by the DPPH radical assay. The results are summarized in Fig. 2. The aminochalcone **5a** shows an IC_50_ value (the antioxidant concentration necessary to decrease the initial amount of DPPH by 50 %) of 4.9 ± 1 µM which is even better than the one for catechol that in the same experimental condition showed an IC_50_ of 5.3 ± 1 µM (see Table 4). The results point out that the presence of the two hydroxyl groups in ring B of **5a** are important for the antioxidant activity of the aminochalcones [10]. For the other two compounds **5** and **6** the IC_50_ is not reached within the concentrations applied. Our values are close to the ones determined for other potent natural flavonoids [36]. Some studies on antioxidant properties of chalcones highlighted a mechanism involving H atom transfer from the phenolic moiety of the ring B [18]. In contrast to these reports no significant free radical scavenging ability is observed for the aminochalcone **5**. This result is comparable to previous antioxidant activity studies performed by Iqbal et al. [37] on a series of 4-aminochalcones and sulfonamide-substituted chalcones, which also did not show a powerful antioxidant activity. Previous studies performed on the trimethoxy chalcone 1-(2,4-dimethoxyphenyl)-3-[3-(4-methoxyphenyl)-1-(1-methylbuta-1,3-dienyl)-1*H*-pyrazol-4-yl]propenone, suggested that the amino group is not involved in the antioxidant mechanism [38]. The measurements reported here evidence only a significant inhibition of DPPH for **5a** that exhibit two phenolic groups in meta and para position on ring B, therefore this would suggest that the mechanism of the H-transfer originate mainly from the phenolic group rather than from the keto-enolic moiety. The two adjacent hydroxyl groups seem to be mainly responsible for the antioxidant activity of the aminochalcone, supporting the mechanism proposed by Hwang et al. [18].

**Table 4:** IC_50_ value of the aminochalcones **5**, **5a**, and **6** for the inhibition of xanthine oxidase and the inhibition of DPPH

Compound	SOD mimic activity IC_50_/μM ± SD^a^	DPPH radical assay IC_50_/μM ± SD^a^
**5**	38.6 ± 1	–
**5a**	37.1 ± 1	4.9 ± 1
**6**	–	–
Caffeic acid	34.1 ± 1	–
Catechol	–	5.3 ± 1

^a^Standard deviation

### Superoxide scavenging ability

**Fig. 3:**
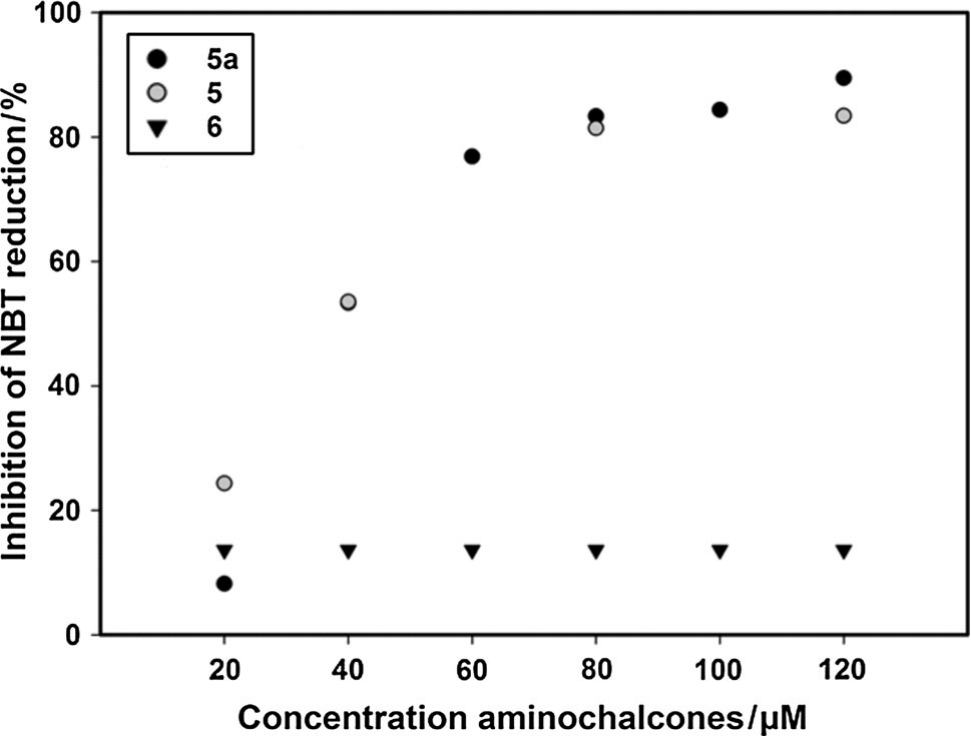
Percentage of inhibition (% In) of superoxide radical formed by xanthine/xanthine oxidase enzyme by different concentrations of 2'-aminochalcones assayed by NBT^2+^ absorption at 560 nm in presence of **5**, **5a**, and **6**. The concentration of the different compounds is expressed as µM

The superoxide radical anions are generated in vitro by a xanthine/xanthine oxidase system causing the reduction of the nitro blue tetrazolium (NBT^2+^) and consequently the determination of the scavenging activity of the chalcones. Applying this method O_2_^**.−**^ reduces the yellow colored (NBT^2+^) to produce the blue formazan, which is measured spectrophotometrically at 560 nm. Antioxidants are scavengers of oxygen free radicals and prevent the purple NBT formation [39]. The compounds **5**, **5a**, and **6** were studied as potential scavengers of oxygen free radicals and the results are summarized in Fig. 3, which illustrates the percentage of inhibition of NBT^2+^ against the concentration of the different synthesized 2'-aminochalcones **5**, **5a**, and **6**. The IC_50_ value, that is the concentration of 2'-aminochalcone necessary to reduce the absorbance of the NBT^2+^ to half of its initial value was calculated to 38.6 ± 1 µM for **5** and 37.1 **±** 1 µM for **5a** (see Table 4). The IC_50_ value of the aminochalcones **5** and **5a** is not so much different to the ones previously found for other flavonoids such as rutin (42.7 µM) and quercetin (42.3 µM) [40]. For comparative purpose, we have also measured the activity of caffeic acid which showed an IC_50_ of 34.1 µM under the same experimental condition. The IC_50_ value is not reached for **6** which showed a very low activity comparable to that one previously found for nobiletin and tangeretin [39]. In previous studies it has been proposed that the reaction takes place by donating the unpaired electron from superoxide radical to the flavonoids [41]. According to this reaction mechanism the presence of at least one hydroxyl group on the B ring seems to be essential for the activity, and would explain the lack of scavenging activity for compound **6**. In fact the percentage of inhibition of superoxide anions is not affected by increasing the concentration of the aminochalcones **6**. Previous studies [42] on superoxide scavenging activity performed on a series of 4′-aminochalcones showed a dose-dependent inhibition of superoxide radicals. In fact it has been observed that the activity increased when the aminochalcones dose level was higher. Interestingly the same aminochalcones possess an electron-releasing group at position 4 of the ring B, therefore this structural characteristic seems to be essential for significant antioxidant activity. Additionally it has been observed that the superoxide scavenging activity is sensitive to the planarity of the molecule [39] present in **5** and **5a**.

## Conclusion

This study reports on the synthesis, characterization, and evaluation of antioxidant activity of three different 2'-aminochalcones. The antioxidant activity was evaluated with two different methods: superoxide scavenging assay and DPPH radical scavenging testing. Compound **5a** having two adjacent hydroxyl groups in the B ring exhibits very high antioxidant activity compared to compounds **5** and **6** based on the DPPH test. The presence of the OH groups in **5** (one in ring B) and **5a** (two adjacent ones in ring B) makes them good candidates for superoxide scavenging activity. Compound **6** having a methoxyl substituent in ring B reveals to be less effective. Therefore, this set of aminochalcones shows a clear correlation between the number and the position of OH groups and their activity. The planar structure evidenced by NMR spectroscopy and X-ray structure analysis seems to be an important prerequisite for the activity of the molecule. The intramolecular hydrogen bond interaction with the adjacent carbonylic oxygen functionality contributes to stabilize the planar structure of the studied chalcones.

## Experimental

Chemicals were purchased from Sigma Aldrich, they were of reagent grade and used without any further purification unless otherwise specified. All NMR spectra were recorded at the NMR core facility 1090 Wien, Währinger Straße 38 of the University of Vienna with a Bruker Avance III 500 MHz NMR spectrometer at 500.32 MHz (^1^H), 125.81 MHz (^13^C), in CDCl_3_, methanol-*d*_4_, or acetone-*d*_6_ at ambient temperature. The splitting of proton resonances in the ^1^H NMR spectra are defined as s = singlet, d = doublet, dd = doublet of doublets, ddd = doublet of doublets of doublets, t = triplet, and m = multiplet. Numbering of carbon atoms and protons refers to Scheme 1. Electrospray ionization mass spectra were recorded at the Mass Spectrometry Center (MSC) of the Chemical Faculty of the University of Vienna on a Bruker Esquire 3000 with an orthogonal ESI source applying MeOH/ACN as solvent. The molecular mass was determined in the positive mode. Elemental analyses were carried out in the Mikroanalytisches Laboratorium of the University of Vienna with a Perkin-Elmer 2400 CHN elemental analyser. The spectrophotometer Shimadzu UV 1800 has been used to recorder the UV–Vis spectra. Spectra were collected in methanol. The infrared spectra were recorded with an infrared spectrometer Bruker Tensor 27 FTIR equipped with a global MIR light source, a KBr beam splitter, and a DLaTGS detector. Sample and background spectra were averaged from 100 scans at 4 cm^−1^ resolution. Undiluted sample powder was pressed on the diamond window of a Harrick MVP 2 diamond ATR accessory. Background spectra were obtained from the empty ATR unit. Data handling was performed with OPUS 5.5 software (Bruker Optik GmbH, 2005). Intensities of reported IR bands are defined as br = broad, s = strong, m = medium, and w = weak.

### 4-(Tetrahydro-2H-pyran-2-yloxy)benzaldehyde (**2**, C_12_H_14_O_3_)

4-Hydroxybenzaldehyde (**1**, 6.60 mmol) and pyridinium *para*-toluenesulfonate (0.16 mmol) were dissolved in 30 cm^3^ of dichloromethane and the solution was mixed at room temperature. Subsequently, 3,4-dihydro-α-pyran (19.73 mmol) was added dropwise. The reaction was stirred for 24 h and the progress was monitored by TLC. Then the mixture was washed with water, dried over Na_2_SO_4_, concentrated under vacuum, and purified by flash column chromatography (hexane/ethyl acetate 7:3) to obtain pure **2**. Yield: 89 %; ^1^H NMR (500 MHz, CDCl_3_): *δ* = 1.50–1.58 (m, 1H, H-10b), 1.60–1.80 (m, 2H, H-9b, 10a), 1.82–1.93 (m, 2H, H-8b, H-9a), 1.96–2.08 (m, 1H, H-8a), 3.64 (m, 1H, H-11b), 3.82–3.93 (m, 1H, H-11a), 5.55 (t, *J* = 3.1 Hz, 1H, H-7), 7.13–7.20 (dd, *J* = 8.9, 2.0 Hz, 2H, H-3, H-5), 7.81–7.87 (dd, *J* = 8.9, 2.0 Hz, 2H, H-2, H-6), 9.97 (s, 1H, C=O) ppm; ^13^C NMR (125 MHz, CDCl_3_): *δ* = 191.1 (C=O), 161.7 (C-4), 132.4 (C-2,6), 131.9 (C-1), 115.9 (C-3,5), 96.1 (C-7), 63.0 (C-11), 30.0 (C-8), 25.3 (C-10), 19.6 (C-9) ppm; HRMS (ESI–MS): *m/z* = 229.0829 ([M+Na]^+^, calcd. for C_12_H_14_O_3_Na 229.0835).

### 3,4-Bis(tetrahydro-2H-pyran-2-yloxy)benzaldehyde (**2a**, C_17_H_22_O_5_)

3,4-Dihydroxybenzaldehyde (**1a**, 6.60 mmol) and pyridinium *para*-toluensulfonate (0.16 mmol) were dissolved in 30 cm^3^ of dichloromethane and the solution was mixed at room temperature. Subsequently, 3,4-dihydro-α-pyran (39.46 mmol) was added dropwise. The reaction was stirred for 24 h and the progress was monitored by TLC. Then the mixture was washed with water, dried over Na_2_SO_4_, concentrated under vacuum, and purified by flash column chromatography (hexane/ethyl acetate 9:2) to obtain **2a** as a mixture of stereoisomers. Yield: 69 %; ^1^H NMR (500 MHz, CDCl_3_): *δ* = 1.58–1.50 (m, 2H, H-10b, 10b′), 1.60–1.80 (m, 4H, H-9b, H-9b′, H-10a, H-10a′), 1. 82–1.93 (m, 4H, H-8b, H-8b′, H-9a, H-9a′), 1.96–2.08 (m, 2H, H-8a, H-8a′) 3.64 (m, 2H, H-11b, H-11b), 3.82–3.93 (m, 2H, H-11a, H-11′a), 5.55 (t, *J* = 2.7 Hz, 2H, H-7, H-7′), 7.05–7.07 (d, *J* = 8.2 Hz, 1H, H-5), 7.25 (s, 1H, H-2), 7.49–7.50 (d, *J* = 8.0 Hz, 1H, H-6), 9.87 (s, 1H, CHO) ppm; ^13^C NMR (125 MHz, CDCl_3_): *δ* = 190.6 (C=O), 153.1 (C-4), 146.9 (C-3), 129.8 (C-1), 123.9 (C-6), 115.8 (C-5), 115.2 (C-2), 98.4 (C-7), 98.9 (C-7′), 63.7 (C-11), 63.3 (C-11′), 30.1 (C-8), 30.1 (C-8′), 25.4 (C-10), 25.2 (C-10′) 19.4 (C-9), 18.8 (C-9′) ppm; HRMS (ESI–MS): *m/z* = 329.1356 ([M+Na]^+^), 307.1534 ([M+H]^+^, calcd. for C_17_H_22_O_5_ 307.1461).

### (*E*)-1-(2-Aminophenyl)-3-[4-(tetrahydro-2*H*-pyran-2-yloxy)phenyl]prop-2-en-1-one (**4**, C_20_H_21_NO_3_)

**2** (4.24 mmol) and **3** (8.48 mmol) were dissolved in 10 cm^3^ of methanol under reflux at 35 °C. Subsequently, a solution of 20 cm^3^ of methanol containing barium hydroxide octahydrate (16.96 mmol) was added dropwise to the reaction mixture. The reaction mixture was stirred for 24 h and the progress was monitored by TLC. Then the mixture was concentrated under vacuum, quenched with 0.1 M HCl and extracted with ethyl acetate. The organic layer was separated, dried over Na_2_SO_4_ and then concentrated under vacuum. The reaction mixture was purified by flash column chromatography (hexane/ethyl acetate 7:3) to obtain pure **4**. Yield: 40 %; ^1^H NMR (500 MHz, CDCl_3_): *δ* = 1.50–1.58 (m, 1H, H-10b), 1.60–1.80 (m, 2H, H-9b, 10a), 1.82–1.93 (m, 2H, H-8b, H-9a), 1.96–2.08 (m, 1H, H-8a), 3.64 (m, 1H, H-11b), 3.82–3.93 (m, 1H, H-11a), 5.55 (t, *J* = 3.1 Hz, 1H, H-7), 6.69–6.71 (m, 2H, H-5′, H-6′), 6.3 (br. s, 2H, NH_2_), 7.08–7.10 (d, 2H, *J* = 8.8 Hz, H-2, H-6), 7.50–7.53 (d, 1H, *J* = 15.4 Hz, H-α), 7.27–7.30 (t, 1H, *J* = 8.1 Hz, H-4′), 7.57–7.59 (d, 2H, *J* = 8.8 Hz, H-5, H-3), 7.71–7.74 (d, 1H, *J* = 15.4 Hz, H-β), 7.86–7.87 (d, 1H, *J* = 8.2 Hz, H-3′) ppm; HRMS (ESI–MS): *m/z* = 346.1426 ([M+Na]^+^), 324.1604 ([M+H]^+^, calcd. for C_20_H_21_NO_3_ 324.1594).

### (*E*)-3-[3,4-Bis(tetrahydro-2*H*-pyran-2-yloxy)phenyl]-1-(2-aminophenyl)prop-2-en-1-one (**4a**, C_25_H_29_NO_5_)

**2a** (4.24 mmol) and **3** (8.48 mmol) were dissolved in 10 cm^3^ of methanol under reflux at 35 °C. Subsequently, a solution of 20 cm^3^ of methanol containing barium hydroxide octahydrate (16.96 mmol) was added dropwise to the reaction mixture. The reaction mixture was stirred for 24 h and the progress was monitored by TLC. Then the mixture was concentrated under vacuum, quenched with 0.1 M HCl and extracted with ethyl acetate. The organic layer was separated, dried over Na_2_SO_4_ and then concentrated under vacuum. The residue yielded the crude chalcone **4a** as a yellow powder and was used for the next step without any further purification. Yield: 39 %; HRMS (ESI–MS): *m/z* = 446.1955 ([M+Na]^+^), 424.2133 ([M+H]^+^, calcd. for C_25_H_29_NO_5_ 424.2118).

### (*E*)-1-(2-Aminophenyl)-3-(4-hydroxyphenyl)prop-2-en-1-one (**5**, C_15_H_13_NO_2_)

**4** (1.26 mmol) was dissolved in 50 cm^3^ of methanol. Subsequently pyridinium *para*-toluensulfonate (0.062 mmol) was added and the reaction was stirred under reflux and the progress was monitored by TLC. Then the reaction mixture was directly concentrated under vacuum and then purified by flash column chromatography (dichloromethane/methanol 9.5/0.5). Yield: 25 %; ^1^H NMR (500 MHz, acetone-*d*_6_): *δ* = 6.60–6.63 (t, *J* = 6.62 Hz, 1H, H-4′), 6.81–6.83 (d, 1H, *J* = 8.2 Hz, H-6′), 6.90–6.92 (d, 2H, *J* = 8.8 Hz, H-2, H-6), 7.04 (s, 2H, NH_2_), 7.24–7.27 (t, *J* = 8.3 Hz, 1H, H-5′), 7.67–7.69 (d, 1H, *J* = 15.4 Hz, H-α), 7.67–7.69 (d, *J* = 8.5 Hz, 2H, H-5, H-3), 7.72–7.75 (d, *J* = 15.4 Hz, 1H, C-β), 8.01–8.03 (d, *J* = 8.2 Hz, 1H, H-3′), 8.83 (s, 1H, OH) ppm; ^13^C NMR (126 MHz, acetone-*d*_6_): *δ* = 190.8 (C=O), 159.5 (C-4), 152.0 (C-2′), 133.7 (C-5′), 130.9 (C-3′), 130.1 (C-α), 127.1 (C-1), 126.8 (C-5, C-3), 120.02 (C-β), 117.6 (C-1′), 115.7 (C-2, C-6), 115.3(C-6′), 114.7 (C-4′) ppm; FT-IR (KBr): $\bar{\nu }$ = 3340, 3042, 3000, 1640, 1600, 1587, 1520, 1326 cm^−1^; UV/Vis (methanol): *λ*_max_ = 240, 342, 399 nm; HRMS (ESI–MS): *m/z* = 262.0832 ([M+Na]^+^), 240.1012 ([M+H]^+^, calcd. for C_15_H_13_NO_2_ 240.1019).

### (*E*)-1-(2-Aminophenyl)-3-(3,4-dihydroxyphenyl)prop-2-en-1-one (**5a**, C_15_H_13_NO_3_)

**4a** (1.26 mmol) was dissolved in 50 cm^3^ of methanol. Subsequently, pyridinium *para*-toluensulfonate (0.062 mmol) was added and the reaction was stirred under reflux at 50 °C and the progress was monitored by TLC. Then the reaction mixture was directly concentrated under vacuum and then purified by column flash chromatography (Sephadex LH-20, dichloromethane/methanol 9.5/0.5). Yield: 20 %; ^1^H NMR (500 MHz, acetone-*d*_6_): *δ* = 6.62–6.64 (t, *J* = 8.2 Hz, H-4′), 6.88–6.90 (d, *J* = 8.2 Hz, 1H, H-6), 6.81–6.83 (d, 1H, *J* = 9.5 Hz, H-6′), 7.03 (s, 2H, NH_2_), 7.16–7.18 (dd, *J* = 10.4, 2.2 Hz, 1H, H-5), 7.24–7.27 (t, 1H, *J* = 8.4 Hz, H-5′), 7.30 (s, 1H, H-2), 7.61–7.58 (d, *J* = 15.4 Hz, 1H, C-β), 7.65–7.68 (d, *J* = 15.4 Hz, 1H, C-α), 8.0–8.02 (d, *J* = 9 Hz, 1H, H-3′), 8.06 (OH), 8.42 (OH) ppm; ^13^C NMR (126 MHz, acetone-*d*_6_): *δ* = 191.0 (C=O), 151.9 (C-2′), 147.6 (C-3), 145.3(C-4), 142.7 (C-β), 133.8 (C-5′), 130.7 (C-3′), 127.7 (C-1′), 121.9 (C-5), 120.0 (C-α), 118.5 (C-1), 116.9 (C-6′), 115.4 (C-4′), 115.3 (C-6), 114.8 (C-2) ppm; FT-IR (KBr): $\bar{\nu }$ = 3325, 1640, 1600, 1573, 1505, 1326 cm^−1^; UV/Vis (methanol): *λ*_max_ = 364, 487 nm; HRMS (ESI–MS): *m/z* = 278.0780 ([M+Na] ^+^), 256.0961 ([M+H]^+^, calcd. for C_15_H_13_NO_3_ 256.0968).

### (*E*)-1-(2-Amino-4,5-dimethoxyphenyl)-3-(4-methoxyphenyl)prop-2-en-1-one (**6**, C_18_H_19_NO_4_)

1-(2-Amino-4,5-dimethoxyphenyl)ethanone (4.24 mmol) and 4-methoxybenzaldehyde (4.24 mmol) were dissolved in 10 cm^3^ of methanol at 35 °C under reflux. Subsequently, a solution of 20 cm^3^ of methanol containing barium hydroxide octahydrate (16.96 mmol) was added dropwise to the reaction mixture. The reaction mixture was stirred for 24 h and the progress was monitored by TLC. Then the mixture was concentrated under vacuum, quenched with 0.1 M HCl and extracted with ethyl acetate. The organic layer was separated, dried over Na_2_SO_4_ and then concentrated under vacuum. The reaction mixture was purified by flash column chromatography (hexane/ethyl acetate 9:2) to obtain pure **6**. Yield: 40 %; ^1^H NMR (500 MHz, methanol-*d*_4_): *δ* = 3.82 (s, 3H, OCH_3_), 3.84 (s, 3H, OCH_3_), 3.86 (s, 3H, OCH_3_), 6.36 (s, 1H, H-6′), 6.96–6.98 (d, *J* = 8.8 Hz, 2H, H-2, H-6), 7.39 (s, 1H, H-3′), 7.56–7.60 (d, 1H, *J* = 15.5 Hz, Hα), 7.60–7.63 (d, *J* = 15.5 Hz, 1H, H-β), 7.68–7.67 (d, *J* = 8.50 Hz, 2H, H-4, H-5) ppm; ^13^C NMR (126 MHz, methanol-*d*_4_): *δ* = 191.6 (C=O), 163.1 (C-4′), 157.8 (C-5′), 151.7 (C-4), 143.3 (C-β), 141.3 (C-1′), 131.3 (C-5, C-3), 129.6 (C-2′), 122.4 (C-α), 115.9 (C-3′), 115.7 (C-2, C-6), 112.2 (C-1), 100.6 (C-6′), 56.4 (C-OCH_3_), 56.2 (C-OCH_3_), 56.1(C-OCH_3_) ppm; FT-IR (KBr): $\bar{\nu }$ = 3386, 3267, 1630, 1563, 1501, 1401, 1342 cm^−1^; UV/Vis (methanol): *λ*_max_ = 244, 323, 412 nm; HRMS (ESI–MS): *m/z* = 336.1206 ([M+Na]^+^), 314.1384 ([M+H]^+^, calcd. for C_18_H_19_NO_4_ 314.1386).

### Crystallographic structure determination

Single-crystal X-ray diffraction data were collected with Bruker diffractometers, equipped with a multilayer monochromator, INCOATEC microfocus sealed tube (*λ* (MoK_α_) = 0.71073 Ǻ), and with a CMOS Photon Detector at 100 K for **6**, and with a APEXII CCD detector for **5** at 200 K, respectively. The data reductions were performed using APEX2 (Bruker Analytical X-ray systems, Madison, 2004) software package. The structure solution was executed using SHELXS [43] with the GUI OLEX2 [44]. The structure refinements were realized with SHELXL [45] embedded in GUI’s OLEX2 [44] and ShelXle [45]. The crystallographic results were proofed with PLATON [46]. OLEX2 [44] calculated modified hkl-files to finalize the refinement process. Both, the original and the modified hkl-files, were uploaded to the CCDC database (http://www.ccdc.cam.ac.uk/) with the accession number 1406883 for **5** and 1406882 for **6**.

### Antioxidant activity (DPPH radical scavenging method)

The antioxidant activity test is conducted according to the method described by Ferrari et al. [47]. 1-*x* cm^3^ of 6 × 10^−5^ mM DPPH radical solution is prepared in methanol and mixed with a variable amount *x* mm^3^ (*x* = 5, 10, 15, 20 mm^3^, etc.) of a methanolic solution containing the 2'-aminochalcone (1.2 mM). The absorbance of the mixture is measured immediately every second up to 30 min at 517 nm at 20 °C. For the baseline a control sample of 1 cm^3^ of methanol was used. The percentage of inhibition of the DPPH radical was calculated for each sample referring to the following formula in accordance to Ref. [47]:

$$\% {\text{In}} = \frac{{A_{0} - A_{t} }}{{A_{0} }} \times 100,$$

where *A*_0_ is the absorbance of the control (DPPH radical) at time 0 and *A*_*t*_ is the absorbance of the mixture DPPH–antioxidant at time *t* (30 min). All the determinations were performed in triplicate and the values of absorbance were corrected considering the factor of dilution. The IC_50_ value was also calculated from the regression line.

### Superoxide dismutase activity (SOD assay using XO/NBT system)

The SOD-like activity is performed with the method described by Ferrari et al. [48] with some modification. Superoxide was generated by enzymatic methods employing a xanthine/xanthine oxidase assay. A mixture containing 50 μM of xanthine, 300 μM of NBT^2+^ (nitro blue tetrazolium) and variable amounts from 0 to 120 μM of 2'-aminochalcone were dissolved in 20 mM phosphate buffer (pH 7.4) in a final volume of 1 cm^3^. The reaction was initiated by the addition of 0.04 unit/cm^3^ of xanthine oxidase. The absorbance changes during the NBT^2+^ formation were monitored spectrophotometrically at 560 nm for 20 min at 25 °C. The percentage of inhibition (% In) of NBT^2+^ formation was calculated according to the equation of the linear regression:

$$A = k_{0} t + k_{1} ,$$

where *A* is the absorbance value, *k*_0_ is the slope of the straight line/the speed of reduction of NBT^2+^, and *t* is the time of reduction, and *k*_1_ is the intercept of the straight line. From this data we were able to calculate the inhibitory ability of the synthesized compounds according to the following formula:

$$\% {\text{In}} = 1 - \frac{{k_{0} }}{{k_{\text{red}} }} \times 100,$$

where *k*_0_ is the slope of the straight line in the presence of the 2'-aminochalcone and *k*_red_ is the speed of reduction of NBT^2+^ by superoxide anions generated by xanthine oxidase at 560 nm in the absence of the antioxidant. The IC_50_ value was also calculated from a linear regression of the observed inhibition against the logarithm of the concentration of the compound.

## Electronic supplementary material

Below is the link to the electronic supplementary material.

Supplementary material 1 (CIF 570 kb)

Supplementary material 2 (PDF 85 kb)

Supplementary material 3 (CIF 4357 kb)

Supplementary material 4 (PDF 166 kb)

Supplementary material 5 (XLS 46 kb)

Supplementary material 6 (DOCX 825 kb)
