# Supplementary material for: Synthesis, structure, and antioxidant activity of methoxy- and hydroxyl-substituted 2'-aminochalcones
Source: Monatsh Chem. 2016 Aug 26;147(10):1747–57. doi: 10.1007/s00706-016-1812-9 (PMC5028401; doi:10.1007/s00706-016-1812-9)

checkCIF/PLATON report

Structure factors have been supplied for datablock(s) chsu253_Iba2

THIS REPORT IS FOR GUIDANCE ONLY. IF USED AS PART OF A REVIEW PROCEDURE FOR PUBLICATION, IT SHOULD NOT REPLACE THE EXPERTISE OF AN EXPERIENCED CRYSTALLOGRAPHIC REFEREE.

No syntax errors found.   CIF dictionary   Interpreting this report

Datablock: chsu253_Iba2

```
Bond precision:  C-C = 0.0043 A         Wavelength=0.71073

Cell:            a=13.1460(13)      b=29.598(3)     c=8.1392(6)
                 alpha=90           beta=90         gamma=90
Temperature:     100 K

                Calculated                 Reported
Volume          3166.9(5)                  3166.9(5)
Space group     I b a 2                    I b a 2
Hall group      I 2 -2c                    I 2 -2c
Moiety formula  C18 H19 N O4               C18 H19 N O4
Sum formula     C18 H19 N O4               C18 H19 N O4
Mr              313.34                     313.34
Dx,g cm-3       1.314                      1.314
Z               8                          8
Mu (mm-1)       0.093                      0.093
F000            1328.0                     1328.0
F000’           1328.68
h,k,lmax        15,35,9                    15,35,9
Nref            2938[ 1586]                2924
Tmin,Tmax       0.993,0.998                0.659,0.745
Tmin’           0.973

Correction method= # Reported T Limits: Tmin=0.659 Tmax=0.745
AbsCorr = MULTI-SCAN

Data completeness= 1.84/1.00      Theta(max)= 25.460

R(reflections)= 0.0403( 2375)     wR2(reflections)= 0.0943( 2924)

S = 1.033                  Npar= 211
```

The following ALERTS were generated. Each ALERT has the format
test-name_ALERT_alert-type_alert-level.
Click on the hyperlinks for more details of the test.

Alert level C

```
STRVA01_ALERT_4_C           Flack parameter is too small
           From the CIF: _refine_ls_abs_structure_Flack   -0.400
           From the CIF: _refine_ls_abs_structure_Flack_su    1.000
PLAT340_ALERT_3_C Low Bond Precision on  C-C Bonds ...............     0.0043 Ang.
```

Alert level G

```
PLAT007_ALERT_5_G Number of Unrefined Donor-H Atoms ..............          2 Report
PLAT032_ALERT_4_G Std. Uncertainty on Flack Parameter Value High .      1.000 Report
PLAT910_ALERT_3_G Missing # of FCF Reflection(s) Below Th(Min) ...          2 Report
PLAT916_ALERT_2_G Hooft y and Flack x Parameter values differ by .       0.20 Check
```

```
   0 ALERT level A = Most likely a serious problem - resolve or explain
   0 ALERT level B = A potentially serious problem, consider carefully
   2 ALERT level C = Check. Ensure it is not caused by an omission or oversight
   4 ALERT level G = General information/check it is not something unexpected

   0 ALERT type 1 CIF construction/syntax error, inconsistent or missing data
   1 ALERT type 2 Indicator that the structure model may be wrong or deficient
   2 ALERT type 3 Indicator that the structure quality may be low
   2 ALERT type 4 Improvement, methodology, query or suggestion
   1 ALERT type 5 Informative message, check
```

It is advisable to attempt to resolve as many as possible of the alerts in all categories. Often the minor alerts point to easily fixed oversights, errors and omissions in your CIF or refinement strategy, so attention to these fine details can be worthwhile. In order to resolve some of the more serious problems it may be necessary to carry out additional measurements or structure refinements. However, the purpose of your study may justify the reported deviations and the more serious of these should normally be commented upon in the discussion or experimental section of a paper or in the "special_details" fields of the CIF. checkCIF was carefully designed to identify outliers and unusual parameters, but every test has its limitations and alerts that are not important in a particular case may appear. Conversely, the absence of alerts does not guarantee there are no aspects of the results needing attention. It is up to the individual to critically assess their own results and, if necessary, seek expert advice.

Publication of your CIF in IUCr journals

A basic structural check has been run on your CIF. These basic checks will be run on all CIFs submitted for publication in IUCr journals (*Acta Crystallographica*, *Journal of Applied Crystallography*, *Journal of Synchrotron Radiation*); however, if you intend to submit to *Acta Crystallographica Section C* or *E*, you should make sure that full publication checks are run on the final version of your CIF prior to submission.

Publication of your CIF in other journals

Please refer to the *Notes for Authors* of the relevant journal for any special instructions relating to CIF submission.

PLATON version of 21/04/2015; check.def file version of 09/03/2015

Datablock chsu253_Iba2 - ellipsoid plot

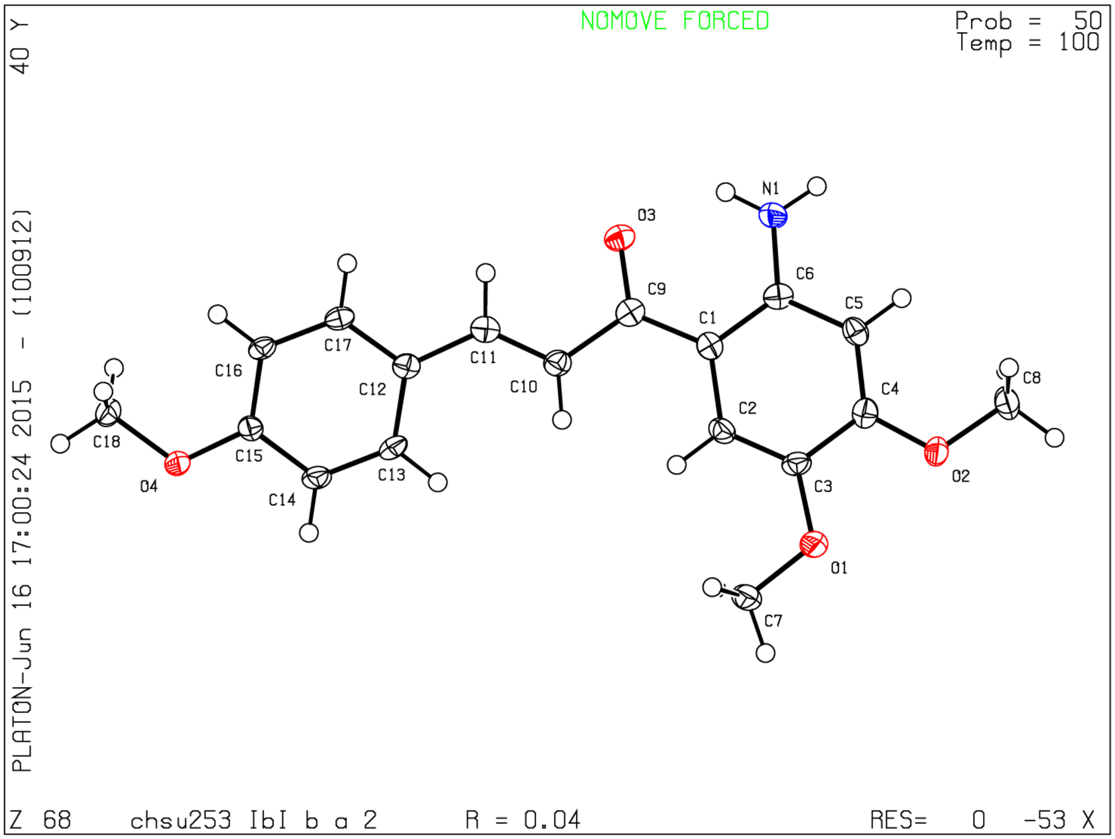

Supplement: Supplementary file 2 — Supplementary material 2 (PDF 85 kb) [file 706_2016_1812_MOESM2_ESM.pdf]
