# Supplementary material for: Synthesis, structure, and antioxidant activity of methoxy- and hydroxyl-substituted 2'-aminochalcones
Source: Monatsh Chem. 2016 Aug 26;147(10):1747–57. doi: 10.1007/s00706-016-1812-9 (PMC5028401; doi:10.1007/s00706-016-1812-9)

checkCIF/PLATON report

Structure factors have been supplied for datablock(s) chsu242lt_0m

THIS REPORT IS FOR GUIDANCE ONLY. IF USED AS PART OF A REVIEW PROCEDURE FOR PUBLICATION, IT SHOULD NOT REPLACE THE EXPERTISE OF AN EXPERIENCED CRYSTALLOGRAPHIC REFEREE.

No syntax errors found. CIF dictionary Interpreting this report

Datablock: chsu242lt_0m

```
Bond precision:  C-C = 0.0031 A          Wavelength=0.71073

Cell:            a=18.877(4)     b=16.644(3)         c=19.387(4)
                 alpha=90        beta=91.781(10)     gamma=90
Temperature:     200 K

                Calculated                 Reported
Volume          6088(2)                    6088(2)
Space group     P 21/c                     P 1 21/c 1
Hall group      -P 2ybc                    -P 2ybc
Moiety formula  C15 H13 N O2               C15 H13 N O2
Sum formula     C15 H13 N O2               C15 H13 N O2
Mr              239.26                     239.26
Dx,g cm-3       1.305                      1.305
Z               20                         20
Mu (mm-1)       0.087                      0.087
F000            2520.0                     2520.0
F000'           2521.15
h,k,lmax        25,22,25                   25,22,25
Nref            15345                      15117
Tmin,Tmax       0.996,0.998                0.746,0.746
Tmin'           0.996

Correction method= # Reported T Limits: Tmin=0.746 Tmax=0.746
AbsCorr = MULTI-SCAN

Data completeness= 0.985          Theta(max)= 28.439

R(reflections)= 0.0579( 8761)     wR2(reflections)= 0.1596( 15117)

S = 1.025                   Npar= 816
```

The following ALERTS were generated. Each ALERT has the format
test-name_ALERT_alert-type_alert-level.
Click on the hyperlinks for more details of the test.

Alert level C

```
PLAT230_ALERT_2_C Hirshfeld Test Diff for    O4C    --  C15C    ..        5.7 su
PLAT410_ALERT_2_C Short Intra H...H Contact  H2A    ..  H10A    ..       1.97 Ang.
PLAT410_ALERT_2_C Short Intra H...H Contact  H2B    ..  H10B    ..       1.97 Ang.
PLAT410_ALERT_2_C Short Intra H...H Contact  H2D    ..  H10D    ..       1.99 Ang.
PLAT420_ALERT_2_C D-H Without Acceptor        N1C    -   H1CA   ..     Please Check
PLAT420_ALERT_2_C D-H Without Acceptor        N1A    -   H1AA   ..     Please Check
PLAT420_ALERT_2_C D-H Without Acceptor        N1E    -   H1EA   ..     Please Check
PLAT906_ALERT_3_C Large K value in the Analysis of Variance ......      4.906 Check
PLAT911_ALERT_3_C Missing # FCF Refl Between THmin & STh/L=  0.600          7 Report
PLAT913_ALERT_3_C Missing # of Very Strong Reflections in FCF ....          1 Note
PLAT918_ALERT_3_C Reflection(s) with I(obs) much smaller I(calc) .          1 Check
```

Alert level G

```
PLAT007_ALERT_5_G Number of Unrefined Donor-H Atoms ..............         15 Report
PLAT720_ALERT_4_G Number of Unusual/Non-Standard Labels ..........         15 Note
PLAT912_ALERT_4_G Missing # of FCF Reflections Above STh/L=  0.600        224 Note
```

---

0 **ALERT level A** = Most likely a serious problem - resolve or explain
0 **ALERT level B** = A potentially serious problem, consider carefully
11 **ALERT level C** = Check. Ensure it is not caused by an omission or oversight
3 **ALERT level G** = General information/check it is not something unexpected

0 ALERT type 1 CIF construction/syntax error, inconsistent or missing data
7 ALERT type 2 Indicator that the structure model may be wrong or deficient
4 ALERT type 3 Indicator that the structure quality may be low
2 ALERT type 4 Improvement, methodology, query or suggestion
1 ALERT type 5 Informative message, check

It is advisable to attempt to resolve as many as possible of the alerts in all categories. Often the minor alerts point to easily fixed oversights, errors and omissions in your CIF or refinement strategy, so attention to these fine details can be worthwhile. In order to resolve some of the more serious problems it may be necessary to carry out additional measurements or structure refinements. However, the purpose of your study may justify the reported deviations and the more serious of these should normally be commented upon in the discussion or experimental section of a paper or in the "special_details" fields of the CIF. checkCIF was carefully designed to identify outliers and unusual parameters, but every test has its limitations and alerts that are not important in a particular case may appear. Conversely, the absence of alerts does not guarantee there are no aspects of the results needing attention. It is up to the individual to critically assess their own results and, if necessary, seek expert advice.

Publication of your CIF in IUCr journals

A basic structural check has been run on your CIF. These basic checks will be run on all CIFs submitted for publication in IUCr journals (*Acta Crystallographica*, *Journal of Applied Crystallography*, *Journal of Synchrotron Radiation*); however, if you intend to submit to *Acta Crystallographica Section C* or *E*, you should make sure that full publication checks are run on the final version of your CIF prior to submission.

Publication of your CIF in other journals

Please refer to the *Notes for Authors* of the relevant journal for any special instructions relating to CIF submission.

PLATON version of 21/04/2015; check.def file version of 09/03/2015

Datablock chsu242lt_0m - ellipsoid plot

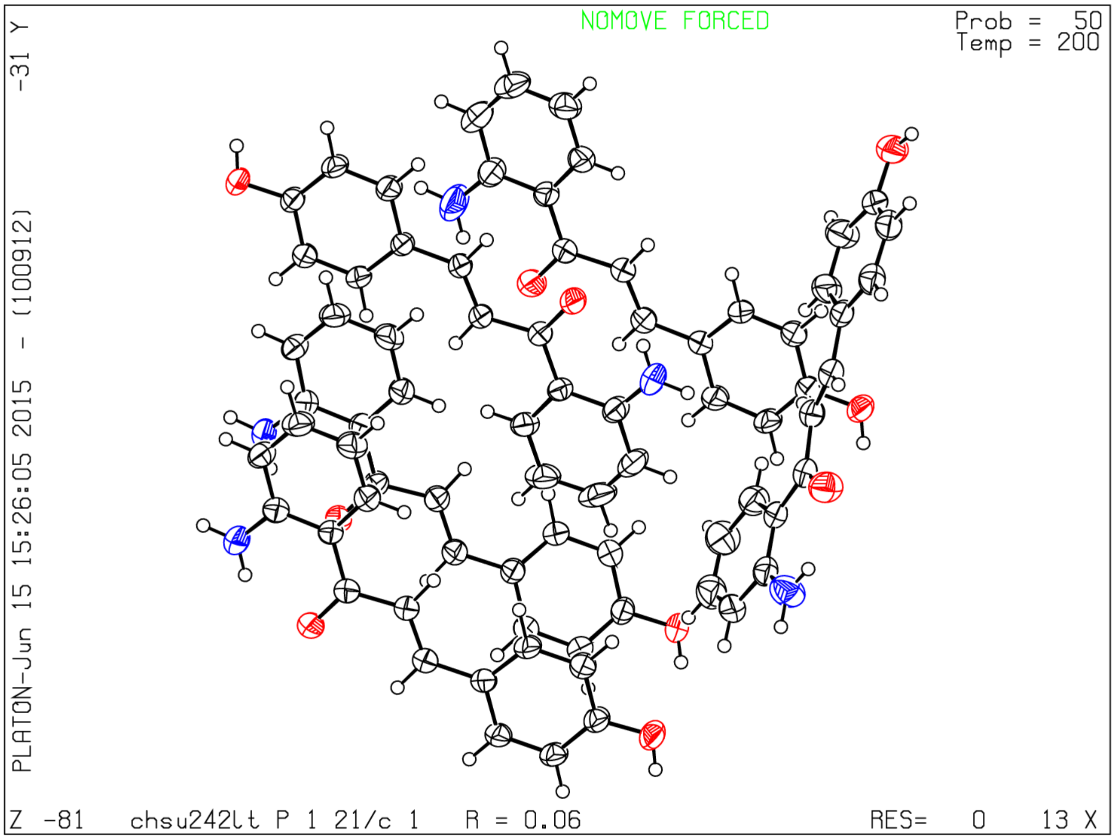

Supplement: Supplementary file 4 — Supplementary material 4 (PDF 166 kb) [file 706_2016_1812_MOESM4_ESM.pdf]
