# Supplementary material for: Synthesis, structure, and antioxidant activity of methoxy- and hydroxyl-substituted 2'-aminochalcones
Source: Monatsh Chem. 2016 Aug 26;147(10):1747–57. doi: 10.1007/s00706-016-1812-9 (PMC5028401; doi:10.1007/s00706-016-1812-9)
Supplement: Supplementary file 6 — Supplementary material 6 (DOCX 825 kb) [file 706_2016_1812_MOESM6_ESM.docx]

Supporting Information

**“Synthesis, structure and antioxidant activity of methoxy and hydroxy substituted 2-aminochalcones”**

Chiara Sulpizio^a^, Alexander Roller^b^, Gerald Giester^c^, Annette Rompel^a^

^a^ Universität Wien, Fakultät für Chemie, Institut für Biophysikalische Chemie, Althanstraße 14, 1090 Wien, Austria

^b^ Universität Wien, Fakultät für Chemie, Zentrum für Röntgenstrukturanalyse, Währinger Straße 42, 1090 Wien, Austria

^c^ Universität Wien, Fakultät für Geowissenschaften, Geographie und Astronomie, Institut für Mineralogie und Kristallographie, Althanstraße 14, 1090 Wien, Austria

# X-ray structures

The structures were solved by direct methods and refined by full-matrix least-squares techniques. Non-hydrogen atoms were refined with anisotropic displacement parameters. Hydrogen atoms were inserted in calculated positions and refined with a riding model respectively as rotating systems. Experimental data can be found in Table 1. Molecular Structures in “Ortep View” are presented in Figures S1 and 2.

| Sample | Machine | Source | Temp. | Detector Distance | Time/ Frame | #Frames | Frame width | CCDC |
| --- | --- | --- | --- | --- | --- | --- | --- | --- |
|  |  |  | [K] | [mm] | [s] |  | [°] |  |
| **5** | Bruker, Kappa ApexII | Mo | 200 (2) | 40 | 70 | 515 | 2 | 1406883 |
| **6** | Bruker D8 | Mo | 100 (2) | 35 | 60 | 776 | 0.5 | 1406882 |

Table S1. Experimental parameters and CCDC-Codes.


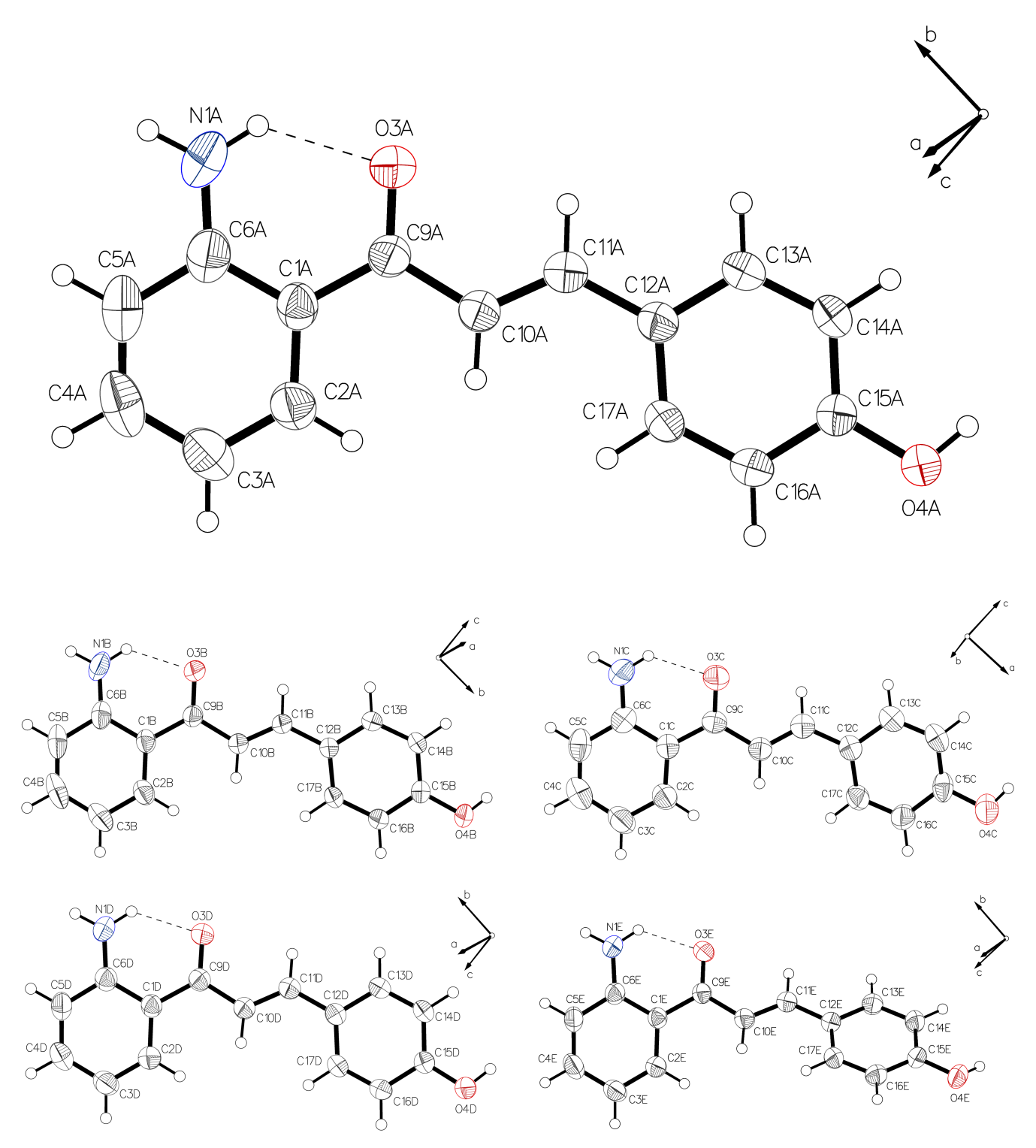


Figure S1. Crystal structure of 5, drawn with 50% displacement ellipsoids. The asymmetric unit is built up by five independent molecules. All of them form one intramolecular hydrogen bond in the range from 2.620 to 2.665 Å for the donor - acceptor distance. The corresponding angle range in the riding hydrogen model is 128.4 to 130.3°. All O4 positions are the basis for intermolecular hydrogen bonds. This is not the case for the positions of N1. The torsion range for N1-C6-C1-C12 is -15.731 to 17.503° and is responsible for the main difference in the 5 molecules from the Asymmetric Unit.


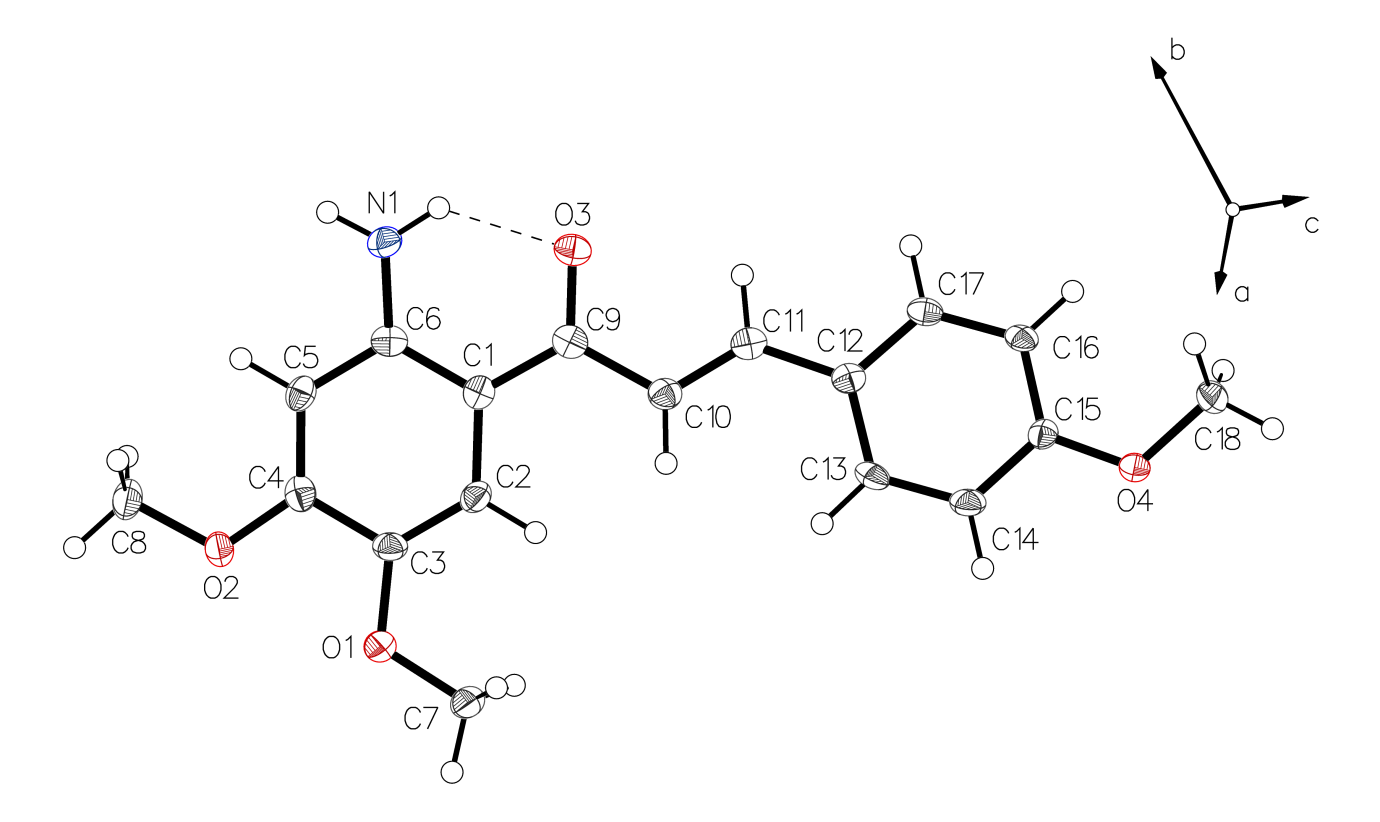


Figure S2. Asymmetric Unit of 6, drawn with 50% displacement ellipsoids. N1 and O3 form one intramolecular hydrogen bond with a donor - acceptor distance 2.584 Å. The corresponding angle in the riding hydrogen model is 130.4. N1 is also responsible for one intermolecular hydrogen bond. The torsion for N1-C6-C1-C12 is -0.702° and is in the range of 6.

# ^1^H NMR spectra


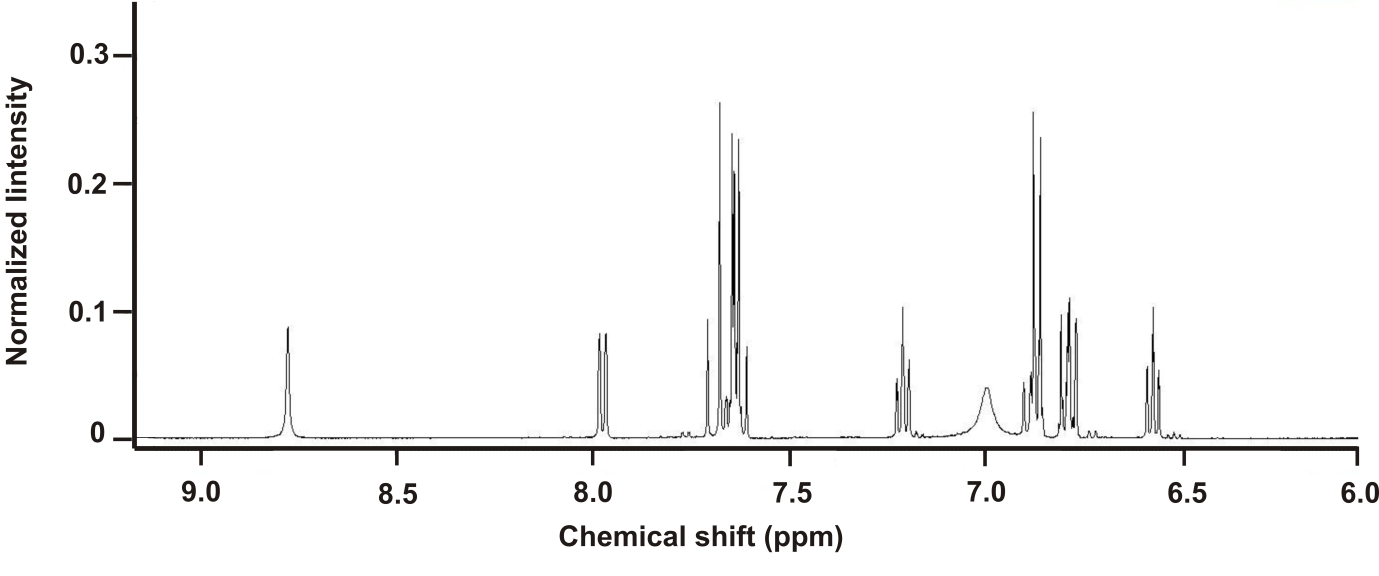


**Figure S3.** ^1^H-NMR in acetone-*d_6_* of (E)-1-(2-aminophenyl)-3-(4-hydroxyphenyl)prop-2-en-1-one (**5**)


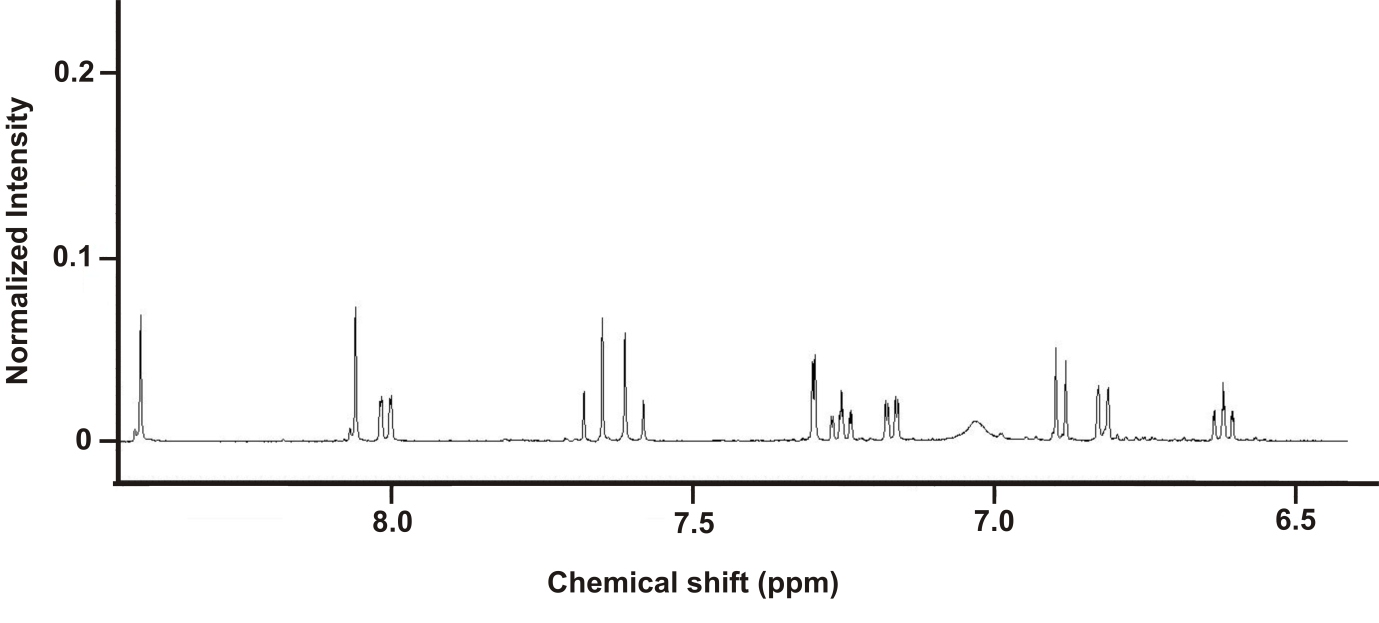


**Figure S4.** ^1^H-NMR in acetone-*d_6_* of E)-1-(2-aminophenyl)-3-(3,4-dihydroxyphenyl)prop-2-en-1-one (**5a**)

**
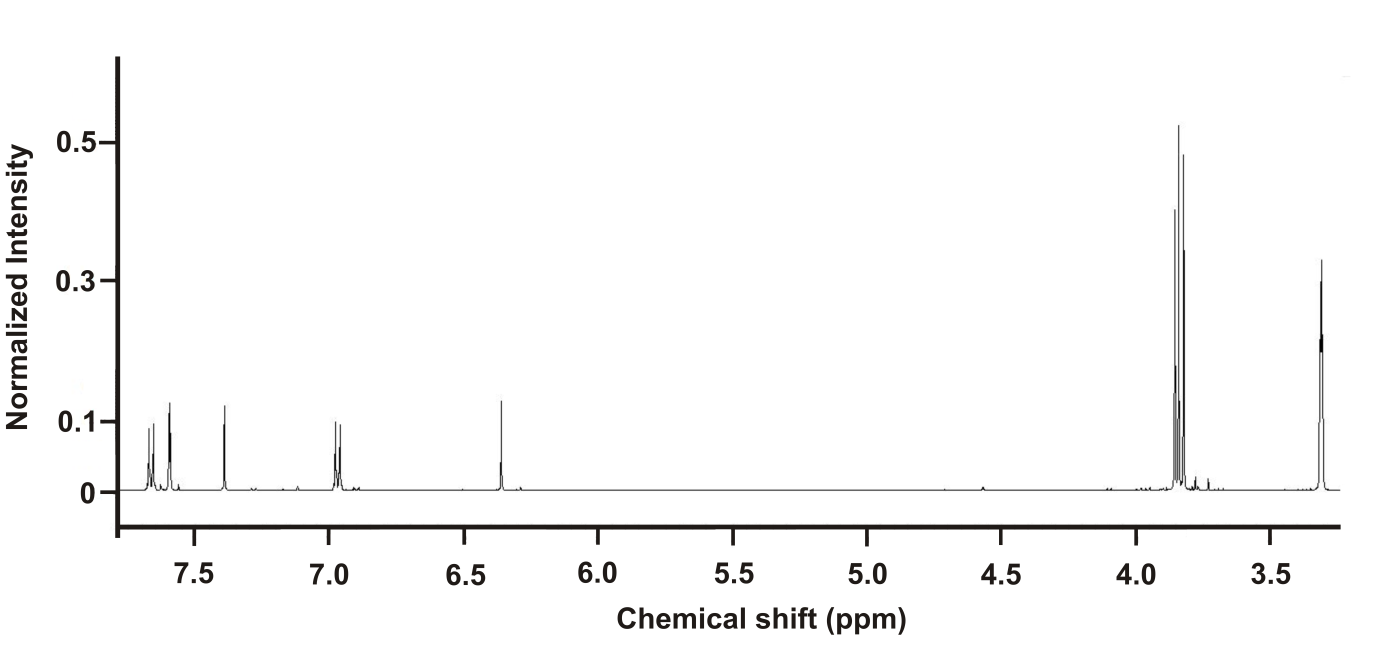
**

**Figure S5.** ^1^H-NMR in MeOD-*d_4_* of (E)-1-(2-amino-4-methoxyphenyl)-3-(3,4-dimethoxyphenyl)prop-2-en-1-one (**6**)

**Scheme 1**
